# Evaluation of an Established Semi-Quantitative Chest CT Scoring System for Assessing the Severity of COVID-19 Pneumonia: What Is Its Diagnostic Value Regarding Patient Outcomes?

**DOI:** 10.3390/diagnostics16183008

**Published:** 2026-09-17

**Authors:** Anna J. Höink, Simon T. Scherfeld, Anna Movlilishvili, Hagen Vorwerk, Michel Eisenblätter, Johann P. Addicks, Eugen Neumann

**Affiliations:** 1 Department of Diagnostic and Interventional Radiology, Klinikum Lippe, Medical School and University Medical Center OWL, Bielefeld University, 32756 Detmold, Germany; 2 Department of Pneumology, Respiratory and Sleep Medicine, Klinikum Lippe Lemgo, Lung Cancer Center Lippe, 32657 Lemgo, Germany

**Keywords:** CT scoring system, COVID-19 pneumonia, AI-based assessment, ICU stay, chest CT

## Abstract

**Background/Objectives**: Investigation of whether visual and AI-based assessments of the severity of COVID-19 pneumonia using an established semi-quantitative chest CT scoring system (Pan score) correlate with laboratory parameters as well as pulmonary function, and of the score’s diagnostic value in predicting the patients’ clinical outcome. **Methods**: This retrospective analysis comprises patients with PCR-confirmed COVID-19 who received a chest CT scan (not more than three days prior to or after the positive PCR test) between 12 August 2020, and 30 December 2022. Each of the five lung lobes was assessed separately using a scoring system ranging from 0 (no pulmonary involvement) to 5 (>75% pulmonary involvement) by a radiology specialist, an experienced resident physician, a medical student, and a dedicated AI-based chest CT software tool. In addition, pulmonary function and laboratory parameters, the duration of ICU stays and of any required mechanical ventilation, as well as the clinical outcome (discharge vs. death) were recorded, and their correlation with the obtained CT score was analysed. Statistical analyses comprised descriptive baseline comparisons using non-parametric tests, bivariate correlation matrices, and ROC curves to assess diagnostic accuracy. Furthermore, multivariable logistic, ordinal, and age-adjusted spline regression models were constructed to calculate odds ratios and estimate predicted probabilities for cumulative ICU and mechanical ventilation duration thresholds. **Results**: In total, 351 consecutive patients with confirmed COVID-19 (223 males [63.5%], 128 females [36.5%]; mean age 67.0 years) were included, all of whom underwent at least one chest CT scan. Compared with patients who were discharged, deceased patients had a significantly (*p* < 0.05) higher mean Pan score (11.7 ± 6.0 vs. 8.8 ± 5.0), higher rates of mechanical ventilation (61.3 vs. 33.0%), and both a higher incidence (42.7 vs. 25.4%) and longer duration (10.5 [6.0–20.0] vs. 6.0 [3.0–10.0] days) of ICU stays. The Pan score showed a moderate and consistent association with the requirement for mechanical ventilation (ρ = 0.49; *q* < 0.001) and the duration of the ICU stay (ρ = 0.43; *q* < 0.001). **Conclusions**: The investigated semi-quantitative CT score is a simple, reliable tool for assessing the extent of COVID-19 pneumonia and can be evaluated both by radiologists and fully automated AI software. While its predictive value for all-cause mortality was only moderate, it showed good performance in predicting the need for and duration of mechanical ventilation, as well as intensive care requirement.

## 1. Introduction

In March 2020, the World Health Organization (WHO) officially declared coronavirus disease 2019 (COVID-19), caused by severe acute respiratory syndrome coronavirus 2 (SARS-CoV-2), a global pandemic. By the end of February 2026, more than 779,000,000 cases of COVID-19 and approximately 7,000,000 deaths had been reported worldwide [1]. Although mortality has steadily declined over time due to vaccination programmes as well as public health and hygiene measures, COVID-19 is still considered endemic in Germany. The high level of population immunity has contributed to a reduction in severe disease courses and long-term sequelae. Nevertheless, further waves of infection are expected, which will predominantly affect elderly individuals and those with pre-existing conditions [2].

Since the onset of the pandemic, chest imaging—particularly computed tomography (CT)—has played a crucial role. It is not only essential for monitoring disease progression and identifying complications, but also for supporting the diagnosis in suspected cases, for example with polymerase chain reaction (PCR) test results that are inconsistent with a clear clinical picture including typical COVID-19 symptoms [3].

On CT imaging, COVID-19 pneumonia most commonly presents with multifocal, predominantly bilateral ground-glass opacities (GGO). These typically exhibit a patchy or geographic distribution and are mainly located in the peripheral and posterobasal regions of the lungs. Consolidations and the so-called crazy-paving pattern are observed less frequently and tend to occur in more advanced stages of the disease [4]. In addition, interlobular septal thickening, vascular enlargement and the halo sign are frequently reported parenchymal findings associated with COVID-19 pneumonia [5].

Quantitative evaluation of lung parenchymal involvement has been shown to be an important factor in predicting disease outcome [6]. One approach is the semi-quantitative CT score proposed by Pan et al., in which each lung lobe is graded according to the extent of involvement (from 0 for no involvement to 5 for >75% involvement). A total score of ≥18—calculated as the sum of all five lobes—is associated with a significantly increased risk of a fatal outcome [7,8]. However, this scoring system only considers the extent of lung involvement and does not account for the specific type of parenchymal abnormalities.

A previous study has already shown that inter-rater agreement on the scores is high, even among readers with varying levels of experience, and that fully automated, AI-based evaluations also produce reliable results [9]. It has also been demonstrated that the score correlates positively with inflammatory parameters, the age of the patients, and the severity of the disease [7].

The aim of this study was to evaluate the diagnostic value of the Pan score with regard to disease severity, i.e., the need for intensive care admission and mechanical ventilation, as well as the patients’ outcomes, and to investigate the extent to which it correlates with various laboratory parameters, the pulmonary function, and the age of the patients. In this way, the aforementioned scientifically validated properties and advantages of the Pan score will be combined to determine whether they can be used beneficially in routine clinical practice to support therapeutic decision-making.

Our hypothesis was that a higher score would be associated with increased inflammatory parameters, a higher likelihood of intensive care admission, a greater probability of a fatal outcome, and poorer pulmonary function—independent of the patients’ age.

## 2. Materials and Methods

For this retrospective study, we included 351 consecutive patients (223 males [63.5%], 128 females [36.5%]; mean age 67.0 years) with PCR-confirmed COVID-19, who underwent chest CT at our hospital between 12 August 2020, and 30 December 2022. The first case of COVID-19 in our district was confirmed in March 2020.

Patients were included based on the following criteria: (a) positive PCR test for COVID-19, (b) chest CT scan performed not more than three days prior or three days after the positive PCR test, (c) age ≥ 18 years. Of the 607 patients initially retrieved, 218 were excluded because their available chest CT examination did not fall within the predefined window of ±3 days relative to the positive PCR result and therefore did not meet inclusion criterion (b). A further 38 patients were excluded owing to motion or breathing artifacts, prior extensive lung surgery, incomplete CT data sets, or other technical difficulties that prevented AI-based evaluation, leaving 351 patients for analysis. Eligibility with respect to the CT–PCR interval was verified using the complete medical record for every case. As this criterion was defined solely by the timing of the examination and was assessed without reference to clinical outcome, the exclusion is unlikely to have introduced outcome-related selection bias.

Approval for this study was granted by the local ethics committee (Medical Association Westphalia-Lippe and University of Münster) and conducted following the principles of the Declaration of Helsinki.

CT images were obtained from the hospital’s picture archiving and communication system (PACS; Centricity^TM^ Universal Viewer, Version 6.0, GE HealthCare Technologies Inc., Chicago, IL, USA) and organised in a dedicated list within the radiological information system (RIS; Centricity^TM^ RIS-i7, Version 7.0.4.4, GE HealthCare Technologies Inc., Chicago, IL, USA).

Laboratory parameters, the results of blood gas analyses and pulmonary function tests, as well as the need for and duration of intensive care unit (ICU) admission and mechanical ventilation, were retrieved from the hospital information system (IS-H, SAP Deutschland SE & Co. KG, Walldorf, Germany). Mechanical ventilation was defined as any documented requirement for ventilatory support, comprising both invasive and non-invasive ventilation (NIV); five patients received NIV only and were included in this endpoint. Ventilation duration was documented for 66 of the 137 ventilated patients; analyses of ventilation duration are therefore based on this subset, whereas patients with documented ventilation but no recorded duration were treated as missing rather than as 0 h.

Chest CT scans were performed using either a 128-slice (SOMATOM Definition Flash, SIEMENS Healthineers AG, Forchheim, Germany) or a 64-slice CT scanner (SOMATOM go.Top, SIEMENS Healthineers AG, Forchheim, Germany). Images were acquired during a single breath-hold from the lung bases to the apices. Scans performed to confirm (or exclude) suspected COVID-19 pneumonia were obtained without intravenous (i. v.) contrast media, while patients scanned for other clinical indications, such as suspected pulmonary embolism or oncologic evaluation, received 40–80 mL of an iodine-containing contrast agent (ACCUPAQUE^TM^-300 or -350, GE HealthCare Buchler GmbH & Co. KG, Braunschweig, Germany) i. v. All scans utilised an automatic exposure control for tube voltage and current. Images were reconstructed using a sharp (pulmonary) and a soft (mediastinal) kernel, with lung images generated at slice thicknesses of 0.8, 1, 2, 3, or 5 mm.

CT images were independently reviewed by a radiology specialist (senior physician), an experienced assistant physician, and a medical student. Readers assessed typical COVID-19 pulmonary features, including GGO, consolidations, and crazy-paving patterns, as well as other abnormalities such as nodules or halo signs. All evaluations were performed blinded to clinical data (laboratory values, blood gas analyses, intensive medical care, etc.) of the patients and to each other’s assessments.

No formal calibration or consensus session was conducted prior to the actual image assessment. The medical student received focused training to accurately identify lung lobes, recognise relevant pathologies, and quantify their extent. The two radiologists, with 14 and 6 years of chest CT experience, were briefed only on the specific pathologies to assess (according to the Fleischner Society’s glossary of terms) and the application of the CT scoring system. As the evaluation of the CT data began in 2023, the definitions of the radiological terms are based on the 2008 version of the Fleischner glossary [10].

Based on the semi-quantitative scoring system described by Pan et al., the extent of parenchymal involvement was assessed separately for each of the five lung lobes. Each lobe was assigned a subjective visual score ranging from 0 to 5 (Table 1). The individual lobar scores were summed to yield a total score ranging from 0 to 25.

**Figure 1:**
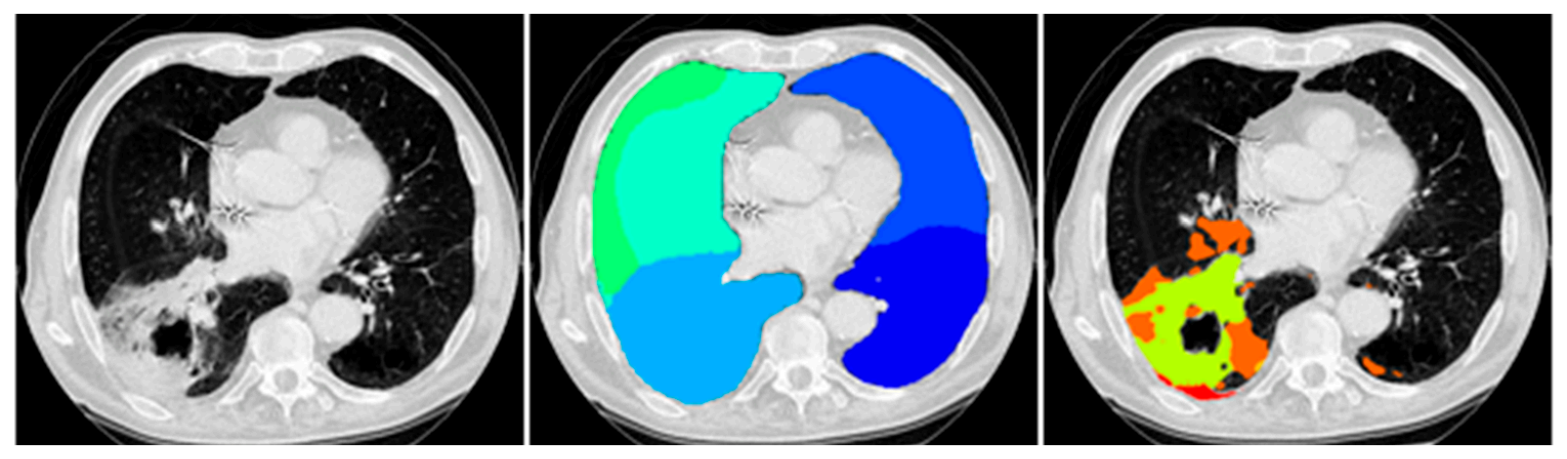
Fully automated evaluation of a chest CT scan by an AI-based software tool (ADVANCE chest CT, contextflow GmbH, Vienna, Austria). (**left**) CT image without any software overlays, (**middle**) automated segmentation of the lung lobes (light green, right upper lobe; turquoise, middle lobe; light blue, right lower lobe; blue, lingula; dark blue, left lower lobe), (**right**) AI-generated detection of circular consolidations in the right lower lobe, marked in yellow, as well as GGO in both lower lobes and in the middle lobe, marked in orange. CT, computed tomography; AI, artificial intelligence; GGO, ground-glass opacities.

In addition to the assessments performed by the three human readers, a fully automated score was generated for each patient using a dedicated AI-based software tool (ADVANCE chest CT, contextflow GmbH, Vienna, Austria; Figure 1). Consequently, four total scores per patient were available for comparative analysis.

The software employs a deep learning-based algorithm applied to lung kernel images to automatically segment the lungs and quantify parenchymal abnormalities. No manual adjustments to the AI-derived results were performed. According to the manufacturer, the algorithm was trained on a multicentric CT dataset; however, detailed information regarding the training process and model architecture is not publicly disclosed.

All statistical analyses and visualisations were performed using R (version 4.x; R Core Team, 2024) [11]. Descriptive statistics were calculated for all variables. Continuous variables are presented as the mean ± standard deviation (SD) or median with interquartile range (IQR), depending on their distribution, while categorical variables are reported as counts and percentages. Group comparisons between survivors and non-survivors were performed using the Wilcoxon rank sum test for continuous variables and the chi-square test or Fisher’s exact test for categorical variables, as appropriate.

Receiver operating characteristics (ROC) curve analyses were conducted to evaluate the discriminatory performance of the Pan score in predicting mortality and the need for mechanical ventilation. The area under the curve (AUC), along with the corresponding confidence intervals (CI), was calculated using the pROC package [12]. Associations between clinical variables, mortality, mechanical ventilation, and ICU length of stay were assessed using Spearman’s rank correlation coefficient (ρ). Rank-based methods were applied consistently throughout because several laboratory parameters, in particular C-reactive protein (CRP) and D-dimer, showed markedly skewed distributions. For the dichotomous variable biological sex (coded male = 1, female = 0), ρ corresponds to a rank-biserial association measure; in addition, sex differences were quantified as odds ratios (OR) with 95% CI derived from logistic regression. To account for multiple testing, *p*-values were adjusted using the Benjamini–Hochberg false discovery rate procedure. Correction was applied separately within each family of tests, i.e., across the 21 tests reported in Table 3 and the tests underlying Figure 9, as these addressed distinct questions. Both unadjusted *p*-values and adjusted *q*-values are reported; associations were considered statistically significant at *q* < 0.05.

Logistic regression models were used to evaluate the association between the Pan score and clinically relevant outcomes, including mechanical ventilation and prolonged ICU stay (≥7 days). Both univariate and multivariable models were fitted, adjusting for potential confounders including age, sex, and laboratory parameters. Ventilation duration was additionally modelled as an ordered categorical outcome using a proportional-odds cumulative logit model (polr, MASS package). The proportional-odds assumption was assessed by a likelihood ratio (LR) test comparing the cumulative logit model with an unconstrained multinomial model. Patients with documented ventilation but no recorded duration were excluded from this analysis rather than assigned to the shortest duration category. Results are reported as OR with 95% CI.

To address heterogeneity in CT acquisition protocols, contrast medium administration and slice thickness were examined as potential confounders. Slice thickness was grouped as ≤1, 2–3, and 5 mm, with 2–3 mm as the reference category, since the individual categories of 0.8 mm (*n* = 5) and 2 mm (*n* = 10) were too small to be analysed separately. Both variables were added to the primary multivariable models as covariates in a sensitivity analysis. In addition, a robustness check was performed in the subgroup of patients who underwent non-contrast CT for suspected COVID-19 pneumonia.

To further explore the relationship between the Pan score and outcome probabilities, generalised linear models with a binomial distribution were fitted. To account for the potentially non-linear effect of age on the outcome, age was modelled using restricted cubic splines (with three degrees of freedom [df]) in all multivariable regression models. This approach provides greater flexibility than assuming a linear relationship between age and the outcome and reduces the risk of residual confounding due to misspecification of the functional form. Predicted probabilities with 95% CI were derived and visualised across the range of Pan scores for cumulative outcome thresholds (e.g., ICU stay ≥ 1, ≥2, ≥3, and ≥7 days; ventilation duration ≥ 24, ≥48, ≥72, and ≥168 h [7 days]).

Data handling and transformation were performed using functions from the tidyverse collection [13]. Model outputs were processed using the broom package [14]. Visualisations were generated using ggplot2 [15], and tables were created using the knitr package [16]. Additional functionality was provided by the splines2, MASS, and nnet packages [17,18,19]. A two-sided *p*-value of <0.05 was considered statistically significant.

Although the order of the individual subsections may vary slightly in some instances, the structure of the paper follows the STROBE guidelines for reporting observational studies [20].

## 3. Results

A total of 351 patients were included in the analysis: 223 males [63.5%], 128 females [36.5%], comprising 276 survivors and 75 non-survivors, corresponding to an overall mortality rate of 21.4%. With respect to the calendar period of inclusion, 38 patients (10.8%) were included in 2020, 109 (31.1%) in 2021, and 204 (58.1%) in 2022. The mean age of the cohort was 67.0 ± 15.9 years, with non-survivors being older on average than survivors (74.0 ± 11.8 vs. 65.1 ± 16.4 years; *p* < 0.001).

The mean Pan score (averaged across all raters) was 9.4 ± 5.3 points and was significantly higher in non-survivors than in survivors (11.7 ± 6.0 vs. 8.8 ± 5.0; *p* < 0.001). Although the Pan scores assigned by the individual readers differed significantly in some cases, the intraclass correlation coefficient (ICC) was high at 0.85 (95% CI: 0.81–0.88), demonstrating excellent inter-reader agreement [9].

Mechanical ventilation was required in 137 of 351 patients (39.0%), while 102 patients (29.1%) were admitted to the ICU. Among those treated in the ICU, the mean length of stay was 7.0 days [3.2–12.0] and was significantly longer in non-survivors compared with survivors (10.5 [6.0–20.0] vs. 6.0 [3.0–10.0] days; *p* < 0.003).

Laboratory parameters showed only modest differences between survivors and non-survivors (Table 2). Laboratory parameters were not available for all patients. Specifically, erythrocyte and leukocyte counts were available for only 349 patients (99.4%) each, CRP values for 297 patients (84.6%), and D-dimer values for only 155 patients (44.2%).

Pulmonary function tests, which could only be performed in selected cases due to hygiene regulations during the COVID-19 pandemic, were conducted in only 16 patients within our cohort. Consequently, the statistical validity of these functional parameters is insufficient for meaningful analyses. Therefore, pulmonary function parameters are not presented in tabular form.

**Figure 2:**
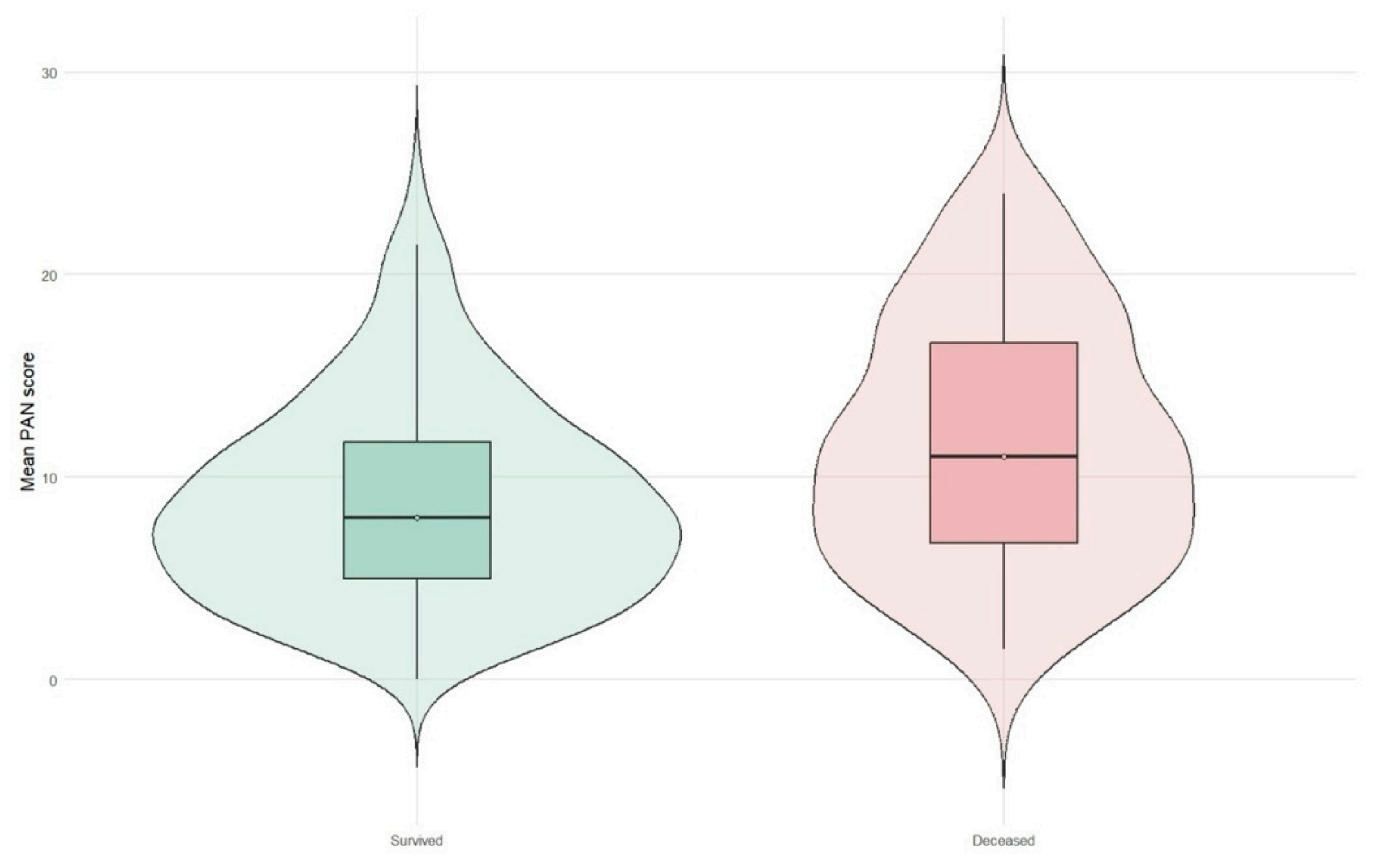
Distribution of Pan scores by outcome across all raters. Violin and box plots demonstrate consistently higher Pan scores in non-survivors (*n* = 75) compared with survivors (*n* = 276) across all rater groups (radiology specialist, assistant physician, medical student, and AI-based software tool). The greatest separation between groups was observed for the AI-based assessment. AI, artificial intelligence.

Pan scores of all four readers were aggregated to derive a single mean Pan score per patient and subsequently stratified by outcome (Figure 2). Non-survivors (*n* = 75) exhibited significantly higher Pan scores than survivors (*n* = 276), with mean values of 11.7 ± 6.0 and 8.8 ± 5.0, respectively (Wilcoxon rank sum test, *p* < 0.001).

**Figure 3:**
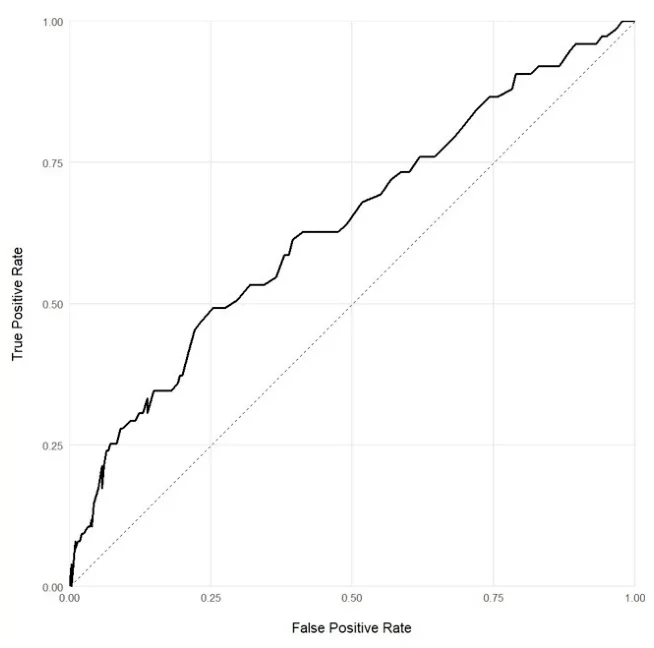
ROC curves for mortality prediction based on Pan scores are shown. Overall, discriminatory performance was limited. The mean Pan score, averaged across all four readers, achieved an AUC of 0.639, indicating only poor to modest ability to differentiate between survivors and non-survivors. The corresponding AUC values for each individual reader and for the AI-based assessment are reported in Supplementary Table S2. ROC, receiver operating characteristic; AUC, area under the curve; AI, artificial intelligence.

However, the ability to discriminate all-cause mortality was limited, as demonstrated by ROC analysis. Discriminatory performance was comparable across the individual readers and the AI-based assessment, with AUC values for mortality ranging from 0.569 to 0.651; corresponding values ranged from 0.751 to 0.788 for mechanical ventilation and from 0.773 to 0.803 for prolonged ICU stay (Supplementary Table S2). The Pan score (overall mean) achieved an AUC of 0.639, indicating only poor to modest predictive performance for all-cause mortality (Figure 3).

Correlation analysis demonstrated that mortality was only weakly associated with the Pan score (ρ = 0.20, *q* = 0.001), age (ρ = 0.23, *q* < 0.001), and selected laboratory parameters (erythrocytes: ρ = −0.17, *q* = 0.006; CRP: ρ = 0.14, *q* = 0.049). In contrast, leukocyte count and D-dimer showed no meaningful correlation with mortality (ρ = 0.03 and 0.05, both *q* > 0.68). The association between biological sex and mortality was nominally significant (ρ = 0.11, *p* = 0.047) but did not withstand correction for multiple testing (*q* = 0.099); in logistic regression, male sex was associated with mortality with an odds ratio of 1.77 (95% CI: 1.02–3.17; *p* = 0.048), whereas no association was observed with mechanical ventilation (OR = 0.90, 95% CI: 0.58–1.41) or prolonged ICU stay (OR = 1.04, 95% CI: 0.58–1.91).

**Figure 4:**
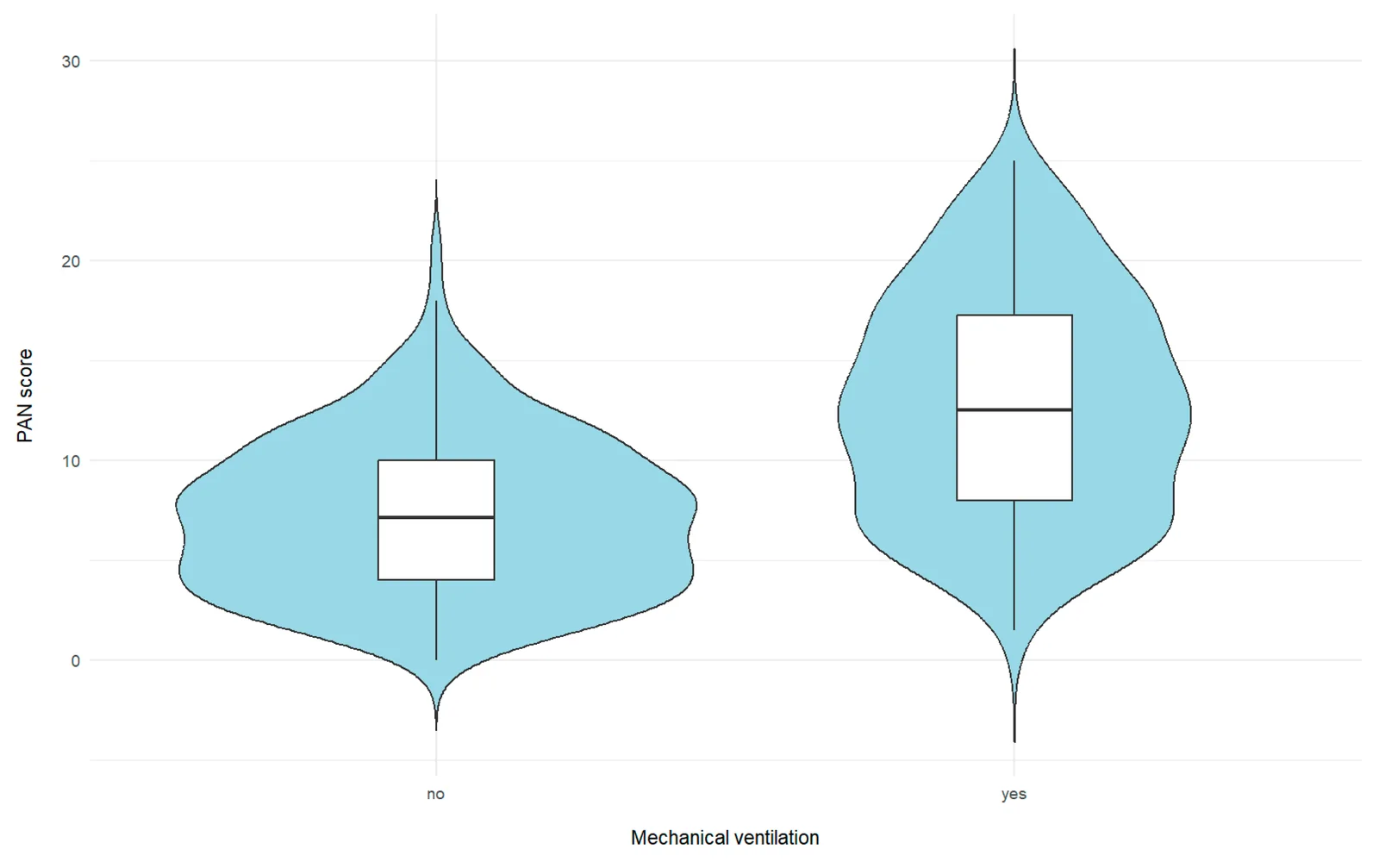
Distribution of Pan scores stratified by mechanical ventilation status. Patients requiring mechanical ventilation (*n* = 137) showed substantially higher Pan scores compared with non-ventilated patients (*n* = 214; Wilcoxon rank sum test, *p* < 0.001).

The Pan score was markedly higher in patients requiring mechanical ventilation (*n* = 137) compared with those who were not ventilated (*n* = 214) (Figure 4). In the correlation matrix, the strongest association was observed between the Pan score and mechanical ventilation (ρ = 0.49, *q* < 0.001), followed by ICU length of stay (ρ =0.43, *q* < 0.001). In contrast, other markers such as CRP (ρ = 0.12, *q* = 0.034) and D-dimer (ρ = 0.12, *q* = 0.140) demonstrated considerably weaker correlations with the need for ventilation, and neither remained significant after correction for multiple testing (*q* = 0.079 and 0.226, respectively) (Table 3).

**Figure 5:**
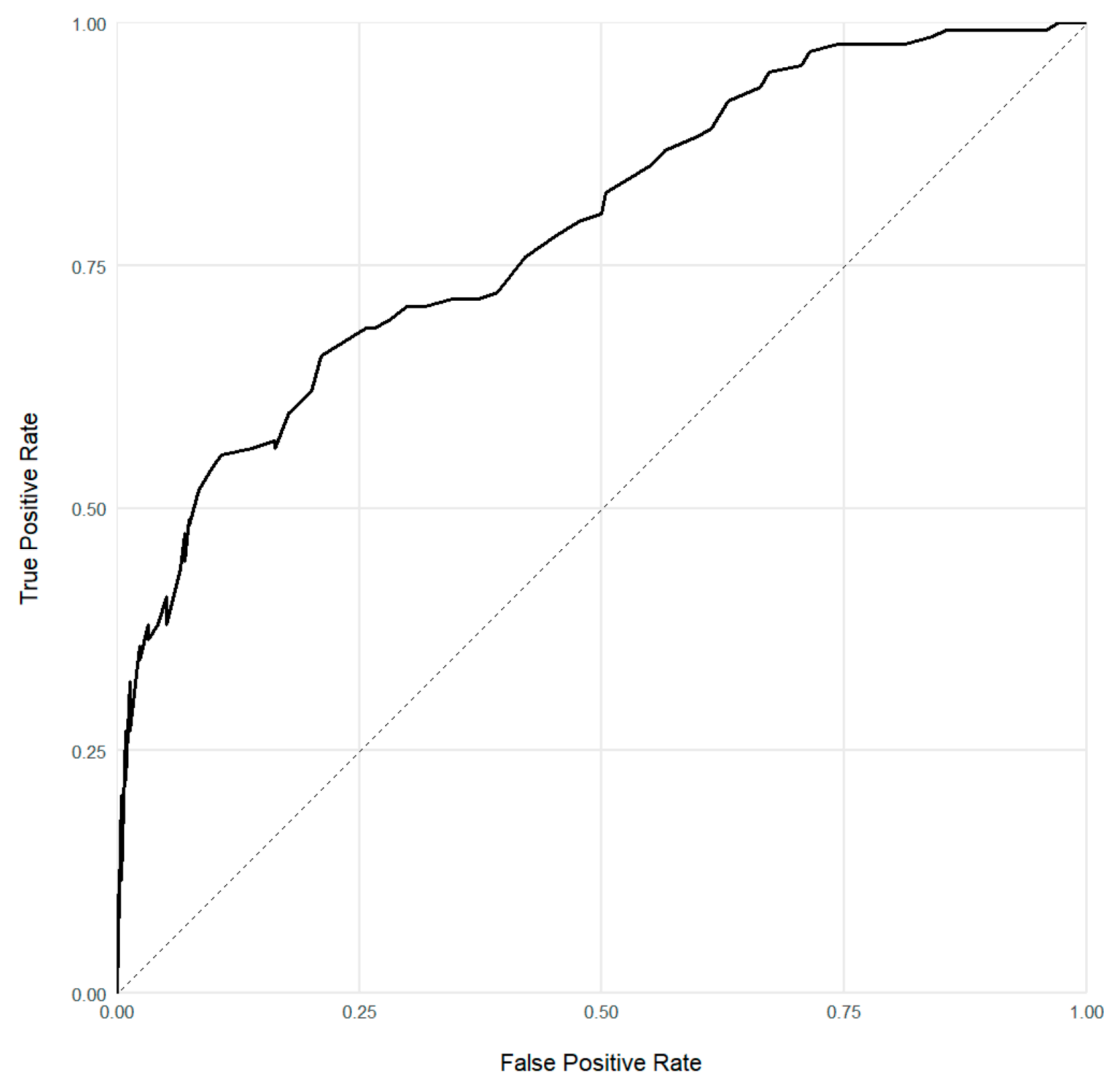
ROC curve for the prediction of mechanical ventilation based on the Pan score. ROC, receiver operating characteristic.

ROC analysis for the prediction of mechanical ventilation using a logistic model based on the Pan score demonstrated good discriminatory performance, with an AUC of 0.788 (95% CI: 0.739–0.837). The optimal probability threshold, determined by Youden’s index, was 0.52, yielding a sensitivity of 0.56, a specificity of 0.89, and an overall accuracy of 0.76 for predicting the need for mechanical ventilation (Figure 5).

**Figure 6:**
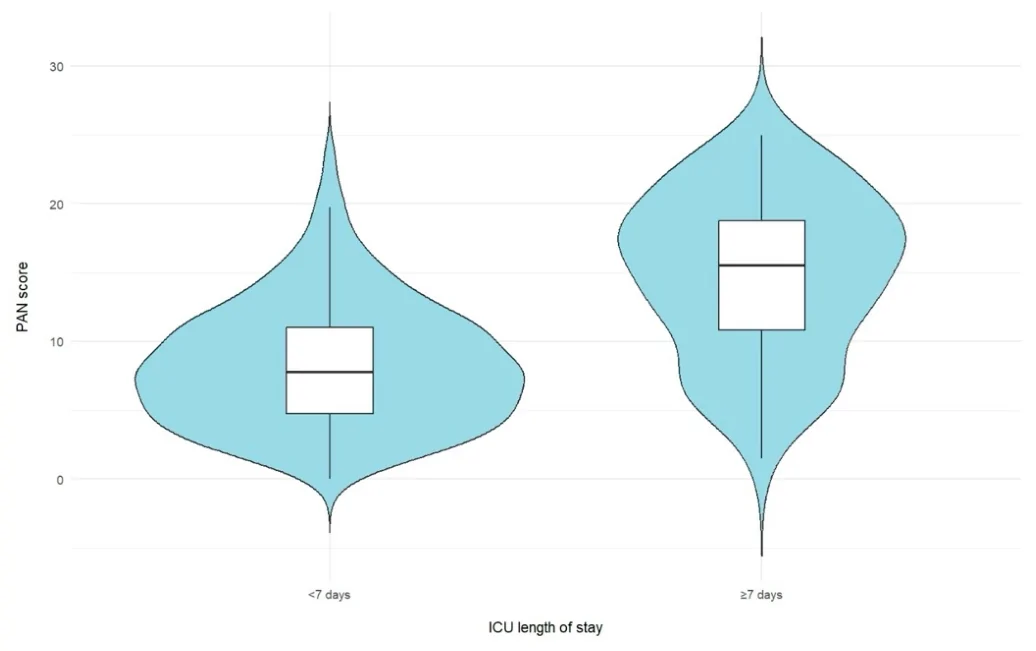
Distribution of Pan scores according to ICU length of stay (<7 vs. ≥7 days). Patients with prolonged ICU treatment (≥7 days) exhibited markedly higher Pan scores than those with shorter ICU stays, in line with the findings from the logistic regression analysis. ICU, intensive care unit.

Patients with prolonged ICU treatment (≥7 days) exhibited markedly higher Pan scores than those with shorter or no ICU stay (Figure 6). The Pan score demonstrated good predictive performance for an ICU stay of ≥7 days, with an AUC of 0.803 (95% CI: 0.733–0.873). The optimal cutoff, determined by Youden’s index, was approximately twelve points (11.9), corresponding to a sensitivity of 0.71, a specificity of 0.81, and an overall accuracy of 0.79 for identifying patients with prolonged ICU stays.

In univariate logistic regression, each one-point increase in the Pan score was associated with a 28% higher odds of requiring mechanical ventilation (*p* < 0.001). The association was essentially unchanged in the primary multivariable model adjusted for age, sex, leukocytes and erythrocytes (OR = 1.29, 95% CI: 1.22–1.38; *n* = 349, 135 events). In sensitivity analyses additionally including CRP (OR = 1.27, 95% CI: 1.19–1.36; *n* = 297) and D-dimer (OR = 1.24, 95% CI: 1.13–1.37; *n* = 138), the estimate remained stable despite a substantial reduction in the analytic sample; laboratory values were available for 297 patients for CRP and 155 for D-dimer, and the smaller analytic samples reflect listwise deletion across all covariates (Table 4).

Similarly, for an ICU stay ≥ 7 days, the univariate odds ratio per one-point increase in Pan score was 1.27 (95% CI: 1.19–1.36; *n* = 346, 56 events; *p* < 0.001), and the association persisted in the primary multivariable model (OR = 1.27, 95% CI: 1.19–1.36; *n* = 344, 55 events; *p* < 0.001). The estimate was likewise unchanged in sensitivity analyses including CRP (OR = 1.28, 95% CI: 1.19–1.38; *n* = 292) and D-dimer (OR = 1.26, 95% CI: 1.12–1.46; *n* = 137). The latter model was based on only 15 outcome events and should be regarded as exploratory (Table 4).

CT examinations were performed without i. v. contrast in 55 patients (15.7%), typically in the setting of suspected COVID-19 pneumonia, and with contrast in 296 patients (84.3%) scanned primarily for suspected pulmonary embolism or oncologic indications. Reconstructed slice thickness was 3 mm in 242 patients (68.9%), 5 mm in 59 (16.8%), 1 mm in 35 (10.0%), 2 mm in 10 (2.8%), and 0.8 mm in 5 (1.4%).

Pan scores differed between acquisition protocols. Patients scanned without contrast had higher Pan scores than those scanned with contrast (median 10.8 [5.8–16.2] vs. 8.1 [5.2–12.0]; *p* = 0.019), consistent with our previous observation [9] and with the fact that these examinations were performed in symptomatic patients with suspected COVID-19 pneumonia. Scores also differed across slice thickness groups (*p* = 0.012), with the lowest median score in examinations reconstructed at ≤1 mm.

These protocol differences did not, however, affect the association between the Pan score and clinical outcomes. After additional adjustment for contrast medium administration and slice thickness, the OR per one-point increase in the Pan score was unchanged for mechanical ventilation (1.29, 95% CI: 1.22–1.38, in all three models) and for prolonged ICU stay (1.27, 95% CI: 1.19–1.36 in the primary model; 1.26, 95% CI: 1.18–1.35 after adjustment for contrast medium; 1.27, 95% CI: 1.19–1.36 after additional adjustment for slice thickness). In a robustness check restricted to the 55 patients scanned without contrast for suspected COVID-19 pneumonia, the estimates were consistent with those of the full cohort (mechanical ventilation: OR = 1.34, 95% CI: 1.17–1.59, 27 events; ICU stay ≥ 7 days: OR = 1.29, 95% CI: 1.14–1.52, 16 events) (Supplementary Table S1). Additional adjustment for the calendar period of inclusion likewise left the estimates unchanged (mechanical ventilation: OR = 1.30, 95% CI: 1.23–1.39; prolonged ICU stay: OR = 1.27, 95% CI: 1.19–1.36).

The Pan score demonstrated a moderate and consistent association with both the need for mechanical ventilation and the duration of ventilatory support. Patients requiring mechanical ventilation had significantly higher Pan scores than those who were not ventilated.

In age-adjusted logistic regression analyses, the Pan score emerged as an independent predictor of mechanical ventilation across all examined duration thresholds. For ventilation lasting ≥ 24 h, each one-point increase in Pan score was associated with a 41% increase in the odds of requiring ventilation (OR = 1.41, 95% CI: 1.30–1.56; *p* < 0.001). Comparable effect sizes were observed for longer ventilation durations (≥48 h: OR = 1.36, 95% CI: 1.26–1.49; ≥72 h: OR = 1.35, 95% CI: 1.25–1.47; ≥7 days: OR = 1.31, 95% CI: 1.21–1.43; all *p* < 0.001).

Discriminatory performance was consistently high across all thresholds, with AUC ranging from 0.859 to 0.886. The highest performance was observed for ventilation ≥ 24 h (AUC 0.886, 95% CI: 0.832–0.940), with only minimal variation across longer ventilation durations.

**Figure 7:**
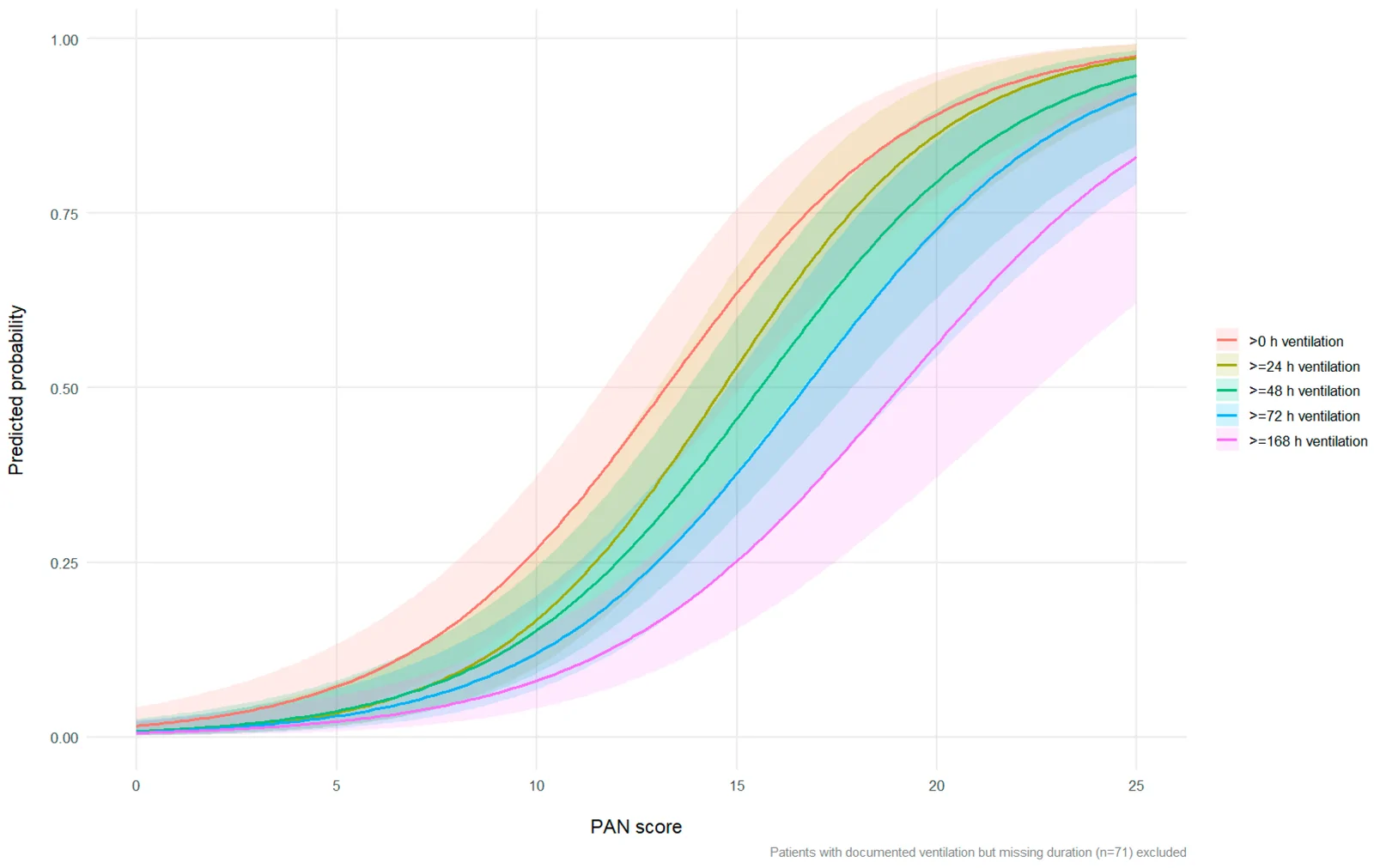
Predicted probabilities of mechanical ventilation across different duration thresholds as a function of the Pan score. The plot shows estimated probabilities for ventilation > 0, ≥24, ≥48, ≥72, and ≥168 h (7 days). Predictions were derived from separate logistic regression models including Pan score and age, with age fixed at the cohort median (69 years). Shaded areas indicate 95% CI. As the thresholds are cumulative (e.g., ≥168 h is a subset of ≥72 h), the curves are not additive. CI, confidence interval.

Ventilation duration was additionally analysed as an ordered categorical outcome using a proportional-odds cumulative logit model with the categories no ventilation (*n* = 214), <24 h (*n* = 13), 24 to <48 h (*n* = 6), 48 to <72 h (*n* = 5), 72 h to <7 days (*n* = 13), and ≥7 days (*n* = 29). The 71 patients with documented ventilation but no recorded duration were excluded from this analysis rather than assigned to the lowest ventilation category, leaving 280 patients. The Pan score showed a significant positive trend (β = 0.295, SE 0.034; OR = 1.34 per one-point increase, 95% CI: 1.26–1.44; *p* < 0.001), indicating a stepwise increase in ventilation duration with rising Pan scores. The proportional-odds assumption was supported by a LR test against an unconstrained multinominal model (LR = 4.49, df = 4; *p* = 0.344) (Figure 7).

Overall, higher Pan scores at baseline CT were associated not only with a greater likelihood of subsequent mechanical ventilation but also with progressively longer durations of ventilatory support.

**Figure 8:**
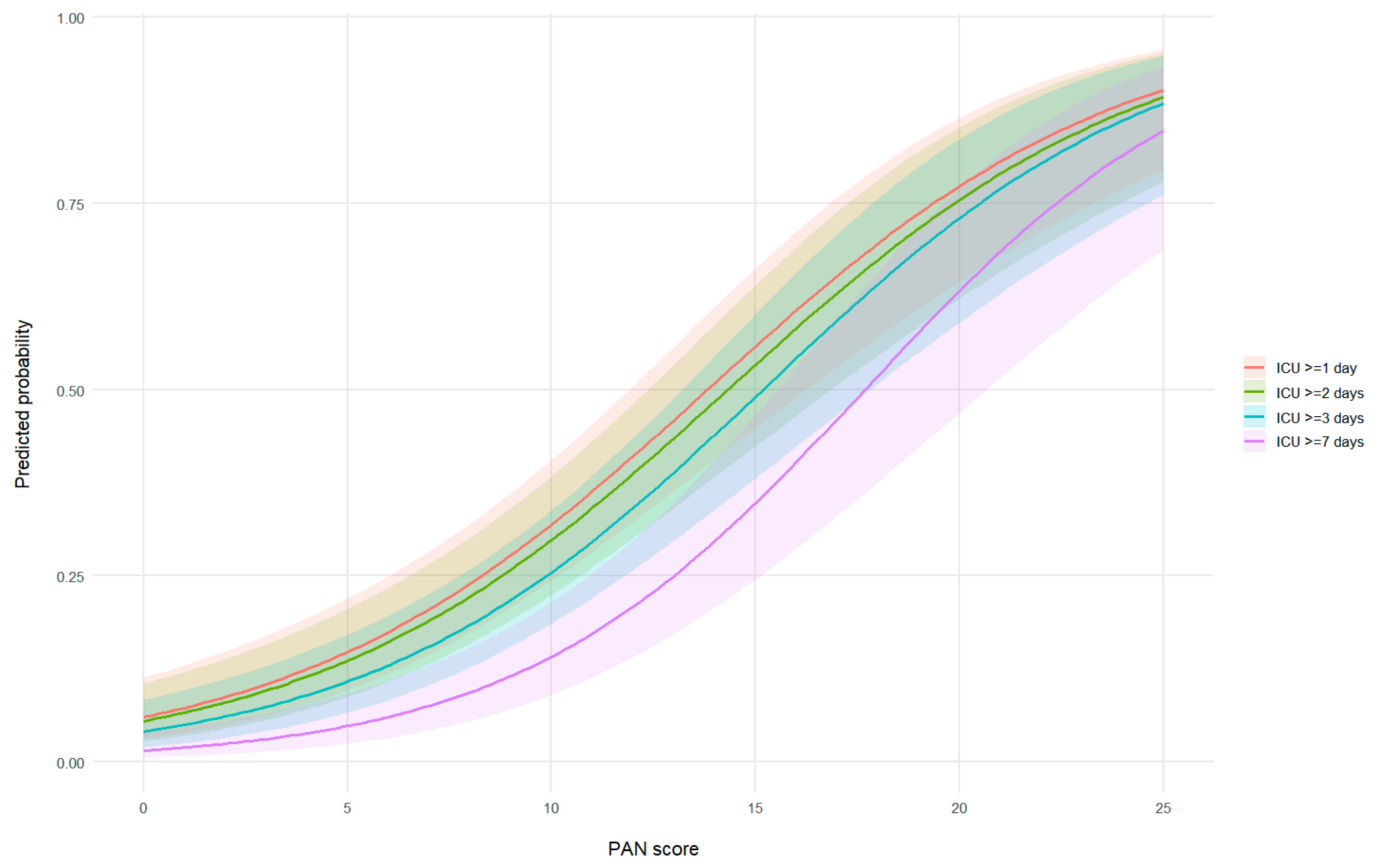
Predicted probabilities of ICU length of stay across different duration thresholds as a function of the Pan score. The figure depicts estimated probabilities of an ICU stay of 0, ≥1, ≥2, ≥3, and ≥7 days. Predictions were derived from separate logistic regression models including Pan score and age, with age fixed at the cohort median (69 years). Shaded bands indicate 95% CI. As the thresholds are cumulative (e.g., ≥7 days is a subset of ≥3 days), the curves are not additive. ICU, intensive care unit; CI, confidence interval.

Predicted probabilities of ICU admission across all duration thresholds (≥1, ≥2, ≥3, and ≥7 days) increased monotonically with the baseline Pan score. When adjusted for the cohort’s median age of 69 years, a Pan score of 15 corresponded to a predicted probability of >50% for an ICU stay of ≥7 days. As the duration thresholds increased, the probability curves showed a consistent rightward shift (Figure 8).

**Figure 9:**
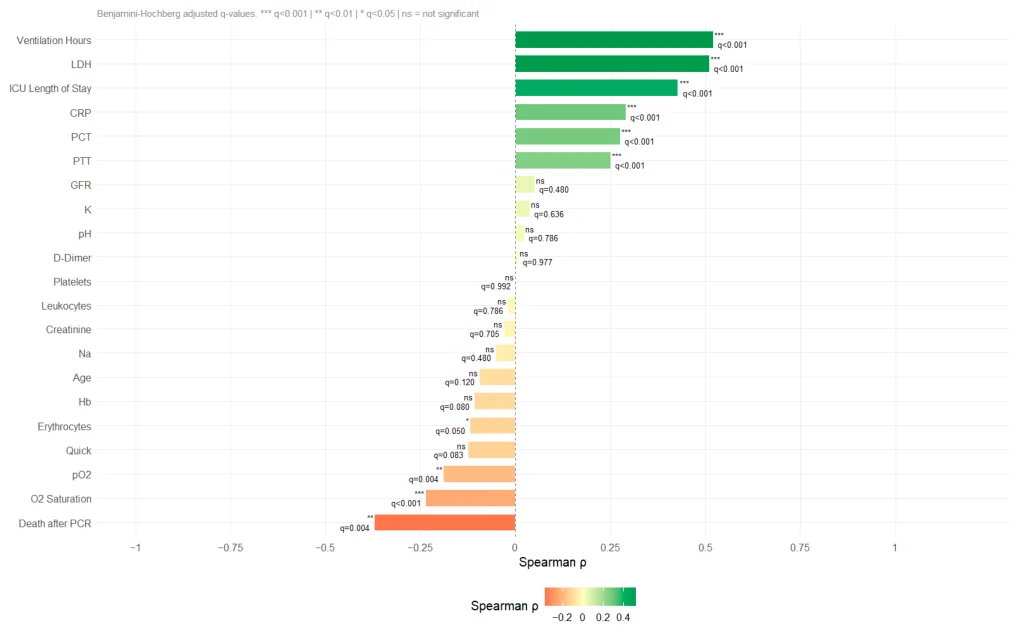
Spearman rank correlation coefficients (ρ) between clinical parameters and the mean Pan score. Bars represent the strength and direction of the monotonic association, ordered from the strongest positive (dark green) to the strongest negative correlation (dark red). Statistical significance is based on *q*-values, i.e., *p*-values adjusted for multiple testing using the Benjamini–Hochberg false discovery rate procedure across all 21 tests shown, and is indicated as *** *q* < 0.001, ** *q* < 0.01, * *q* < 0.05, and ns = not significant. Moderate positive correlations were observed for the duration of mechanical ventilation, LDH, ICU length of stay, CRP, PCT, and PTT, whereas respiratory parameters (oxygen saturation and pO_2_) and the interval from PCR confirmation to death showed inverse correlations. The number of available observations differs between parameters and is reported in Supplementary Table S3. LDH, lactate dehydrogenase; ICU, intensive care unit; CRP, C-reactive protein; PCT, procalcitonin; PTT, partial thromboplastin time; GFR, glomerular filtration rate; K, potassium; pH, potential of hydrogen; Na, sodium; Hb, haemoglobin; pO_2_, partial pressure of oxygen; PCR, polymerase chain reaction.

To further characterise these findings, a bivariate correlation analysis was performed between the mean Pan score across all readers and selected clinical, laboratory, and outcome parameters (Figure 9). Significant positive monotonic correlations were observed between the mean Pan score and markers of systemic inflammation and tissue injury, most markedly lactate dehydrogenase (LDH; ρ = 0.51, *q* < 0.001), followed by CRP (ρ = 0.29, *q* < 0.001), procalcitonin (ρ = 0.28, *q* < 0.001), and partial thromboplastin time (PTT; ρ = 0.25, *q* < 0.001). In addition, higher Pan scores were associated with increased healthcare resource utilisation, as reflected by prolonged duration of mechanical ventilation (ρ = 0.52, *q* < 0.001) and longer ICU stays (ρ = 0.43, *q* < 0.001). In contrast, significant negative correlations were found with respiratory parameters, including oxygen saturation (ρ = −0.24, *q* < 0.001) and arterial pO_2_ (ρ = −0.19, *q* = 0.004). Among patients who died, higher Pan scores were associated with a shorter interval between PCR confirmation and death (ρ = −0.37, *q* = 0.004; *n* = 71). No significant correlations were identified for D-dimer levels, leukocyte count, platelet count, or estimated glomerular filtration rate (eGFR). Notably, the Pan score showed no significant correlation with patient age (ρ = −0.10, *q* = 0.120), indicating that the score captures information independent of age. Of the twelve associations reaching nominal significance, ten remained significant after Benjamini–Hochberg correction; haemoglobin and prothrombin time did not withstand adjustment.

## 4. Discussion

The aim of this study was to investigate the extent to which the semi-quantitative CT scoring system proposed by Pan et al. [8] for assessing the severity of COVID-19 pneumonia can predict the clinical course of affected patients. This involved assessing the correlation of the score with various laboratory parameters and the patients’ pulmonary function, as well as evaluating its ability to predict the need for mechanical ventilation, admission to intensive care, and survival.

The Pan score was determined independently by three different readers with varying levels of radiological experience, as well as by an AI-based software application, as described previously [9]. For the present study, the mean score of all four readers was used for comparison with other parameters in each case. Compared with other studies investigating the outcome of COVID-19 patients or the correlation of SARS-CoV-2-associated changes on chest CT with various clinical parameters [6,7,21,22,23,24], the patient cohort examined in our study (*n* = 351) was considerably larger.

The overall mortality rate in our cohort was 21.4%, which is consistent with the rates reported in other studies, ranging from 15.4 to 33.1% [6,7,23,24]. The COVID-positive patients who died were, on average, significantly older than those who were discharged from hospital alive. In addition, the deceased patients showed significantly higher Pan scores (11.7 ± 6.0 vs. 8.8 ± 5.0). These findings are consistent with the observations of Li et al., who demonstrated that the mortality rate in COVID-19 infections increases with higher CT scores [24]. Although they used an earlier CT scoring system to semi-quantitatively assess the extent of pulmonary involvement, it employed the same threshold values as the Pan score and, in terms of its assessment—based on separate evaluation of different lung segments—was also comparable to it [25].

However, when interpreting the mortality rate, it should be considered that, due to the need to exclude a considerable number of patients from the study population because they did not fully meet the strict inclusion criteria (see below), potential bias in the mortality rate cannot be ruled out. Furthermore, these exclusions may have influenced the distribution of clinical courses.

Of the laboratory parameters examined, only erythrocytes (4.4 vs. 3.9/pL) and CRP (4.6 vs. 7.3 mg/dL) showed significant differences between survivors and non-survivors; leukocytes and D-dimer did not differ significantly.

In our study, the predictive power of the Pan score with regard to all-cause mortality was only moderately pronounced. This contradicts the findings of Francone et al., who observed that the risk of death significantly increased at a CT score of ≥18, and the observations of Szabó et al., who demonstrated that death was significantly associated with higher CT scores [6,7].

By contrast, the Pan score showed good discriminatory ability in predicting the need for mechanical ventilation, and furthermore, the score was moderately correlated with the duration of mechanical ventilation. The proportion of patients requiring mechanical ventilation was significantly higher among non-survivors than among survivors (61.3 vs. 33.0%), and patients who required mechanical ventilation exhibited significantly higher Pan scores. Each additional point in the Pan score was associated with a 28% increase in the odds of requiring mechanical ventilation, and with regard to ventilation duration, the score showed a significant positive trend, with a stepwise increase in the duration of mechanical ventilation as scores rose. For ventilation lasting ≥ 24 h, each additional point in the Pan score corresponded to a 41% increase in the odds of requiring ventilation.

The Pan score also showed good predictive performance in assessing the need for intensive care admission in patients with COVID-19 pneumonia, especially for ICU stays of ≥7 days. Both the rate of ICU admissions (42.7 vs. 25.4%) and the length of ICU stays (10.5 vs. 6.0 days) were significantly higher among non-survivors than among survivors. At a threshold of 11.9 points, the Pan score demonstrated an accuracy of 0.79 in identifying patients requiring prolonged intensive care. After adjustment for the cohort’s median age of 69 years, a Pan score of 15 was associated with a predicted probability of >50% for an ICU stay of ≥7 days.

These findings should be further investigated in future studies with regard to the long-term outcomes of patients who have survived COVID-19 pneumonia. In particular, it would be highly relevant to determine whether the initial Pan score can provide information on chronic post-infectious changes in the lung parenchyma, especially given that the frequency and severity of long-term pulmonary abnormalities have already been shown to correlate with the severity of the initial infection [26].

Among the laboratory parameters examined, only weak overall correlations were observed with the endpoints of mortality, mechanical ventilation, and intensive care admission. CRP was weakly correlated with patient mortality (ρ = 0.14) and ICU length of stay (ρ = 0.17), and erythrocyte count showed weak inverse correlations with both endpoints (ρ = −0.17 and −0.16, respectively); these associations persisted after correction for multiple testing. By contrast, leukocyte count and D-dimer showed no meaningful correlation with any endpoint once multiple testing was accounted for. Pulmonary function parameters showed no meaningful correlation with the above-mentioned endpoints. Furthermore, pulmonary function tests were performed in only a small number of patients in our cohort, precluding any reliable statistical analysis. Patient age also demonstrated only a weak correlation with mortality (ρ = 0.23).

The main limitation of our study is its retrospective design. As a result, we were confronted, on the one hand, with different CT protocols, as patients with a confirmed COVID-19 infection predominantly underwent non-contrast chest CT to assess the extent of pulmonary involvement [27,28], whereas others received contrast-enhanced CT scans (e.g., for oncological staging or suspected pulmonary embolism), where COVID-19 was not the primary clinical concern. In the present cohort, the latter group predominated: contrast-enhanced examinations were performed in 296 patients (84.3%) and unenhanced examinations in 55 (15.7%). Furthermore, the reconstructed CT images used for our analyses had varying slice thicknesses, due to the slightly different imaging protocols still in use at the two sites of our hospital at that time.

We had already confirmed in a previous study the hypothesis that patients undergoing non-contrast CT for symptomatic COVID-19 infection exhibit higher Pan scores than, for example, patients in oncological follow-up [9]. The present cohort is consistent with this observation: patients scanned without contrast had higher Pan scores than those scanned with contrast (median 10.8 vs. 8.1; *p* = 0.019), and scores also differed across slice thickness groups (*p* = 0.012). However, additional adjustment for both contrast medium administration and slice thickness left the association between the Pan score and clinical outcomes essentially unchanged, and a robustness check restricted to non-contrast examinations performed for suspected COVID-19 pneumonia yielded consistent estimates. Protocol heterogeneity therefore appears to influence the absolute level of the score rather than its relationship with clinical outcomes.

On the other hand, we had to deal with the fact that the laboratory parameters of interest were partially or incompletely documented, and pulmonary function testing was performed in only a small number of patients. The latter is explained by the fact that, during the COVID-19 pandemic, pulmonary function testing was generally recommended only in emergency situations for hygiene reasons [29]. The corresponding attrition rate of approximately 42% was, however, driven predominantly by an eligibility criterion rather than by data availability: 218 of the 256 excluded patients did not have a CT examination within the predefined window of ±3 days relative to the positive PCR result. As this criterion depends solely on the timing of the examination and was assessed without reference to clinical outcome, it is unlikely to have introduced outcome-related selection bias. Nevertheless, there remains a residual risk that the timing of the CT examination may have had some influence on the score’s diagnostic value, as patients whose condition deteriorated very rapidly, as well as those who were discharged from inpatient care at an early stage, may systematically not have undergone the examination within the specified time window.

As the COVID-19 vaccination programme in Germany commenced during the study period and given that some of the patients may already have experienced a second or possibly even a third COVID-19 infection at the time of study inclusion, it can be assumed that these factors may have influenced both the severity of the observed COVID-19 pneumonia and mortality, independently of the Pan score. The same applies to the fact that, over the observation period of almost two and a half years, which spans the wild-type, Alpha, Delta and Omicron eras, different viral variants emerged and new treatment options were established [2]. For example, Tsakok et al. demonstrated that patients infected with the Omicron variant had better outcomes than those infected with the Delta variant [30]. Reassuringly, the association between the Pan score and clinical outcomes was unchanged after additional adjustment for calendar period (mechanical ventilation: OR = 1.30, 95% CI: 1.23–1.39; prolonged ICU stay: OR = 1.27, 95% CI: 1.19–1.36), indicating that the predictive value of the score was stable across the pandemic phases covered. Unfortunately, vaccination status and, where applicable, previous COVID-19 infections were not routinely documented at the time of admission, meaning that we were unable to perform retrospective subgroup analyses.

In recent years, the number of COVID-19 infections has declined markedly due to established vaccination programmes and the population immunity acquired through prior infection, meaning that COVID-19-related pulmonary changes are now observed only rarely. Nevertheless, further waves of transmission are still considered possible, and severe disease courses may continue to occur, particularly among older individuals and those with certain pre-existing conditions. Furthermore, antigenic shifts remain possible, which could temporarily lead to increased viral circulation and a higher disease burden once again [2].

Therefore, the assessment of semi-quantitative CT scores and their predictive value remains relevant, as these scoring systems may be adapted in the future for evaluating other pulmonary infectious diseases. Moreover, the observation that higher Pan scores are associated with increased healthcare resource utilisation may also be relevant when further evaluating therapeutic strategies and their effectiveness. The previously demonstrated feasibility of reliably determining the score fully automatically using an AI-based approach also provides a basis for further studies investigating the economic benefits of AI-based solutions, for example by enabling a more efficient use of limited human resources within the healthcare system, particularly in the event of a future pandemic or endemic outbreak.

## 5. Conclusions

The semi-quantitative CT score investigated is a simple and reliable diagnostic tool for assessing the extent of COVID-19 pneumonia, which can be determined both subjectively by radiologists with varying levels of experience and fully automatically by AI-based software. The score showed only moderate predictive performance for all-cause mortality but performed well in predicting the need for and duration of mechanical ventilation, as well as the intensive care requirement in affected patients.

## Figures and Tables

**Table 1:** A semi-quantitative chest CT scoring system, as described by Pan et al. [8], is used to assess the proportion of lung parenchyma affected by pathological changes in COVID-19 pneumonia. CT, computed tomography; COVID-19, coronavirus disease 2019.

Score	Extent of Involvement of Each Lung Lobe
0	no involvement
1	<5%
2	5–25%
3	26–49%
4	50–75%
5	>75%

**Table 2:** Values of the different variables in the overall cohort, the cohort of survivors, and the cohort of non-survivors. Data are presented as mean ± SD or as median with IQR (in square brackets). Corresponding *p*-values are also reported. IQR, interquartile range; AI, artificial intelligence; ICU, intensive care unit; CRP, C-reactive protein; pL, picolitre; nL, nanolitre; mg, milligramme; dL, decilitre; L, litre.

Variable	Overall Cohort (*n* = 351)	Survivors (*n* = 276)	Non-Survivors (*n* = 75)	*p*-Value
Age [years]	67.0 ± 15.9	65.1 ± 16.4	74.0 ± 11.8	<0.001
Pan score				
total	9.4 ± 5.3	8.8 ± 5.0	11.7 ± 6.0	<0.001
radiology specialist	8.1 ± 5.7	7.4 ± 5.2	10.7 ± 6.6	<0.001
assistant physician	11.2 ± 6.1	10.8 ± 5.9	12.5 ± 6.5	0.062
medical student	8.0 ± 6.4	7.2 ± 5.8	11.0 ± 7.7	<0.001
AI	10.3 ± 4.9	9.7 ± 4.6	12.5 ± 5.4	<0.001
ICU				
rate [%]	29.1	25.4	42.7	0.002
length of stay [days]	7.0 [3.2–12.0]	6.0 [3.0–10.0]	10.5 [6.0–20.0]	0.003
Mechanical ventilation rate [%]	39.0	33.0	61.3	<0.001
Laboratory parameters				
Erythrocytes [/pL]	4.4 [3.7–4.9]	4.4 [3.9–4.9]	3.9 [3.4–4.7]	0.001
Leukocytes [/nL]	7.5 [5.6–10.4]	7.5 [5.7–10.1]	7.9 [5.2–10.9]	0.558
CRP [mg/dL]	5.0 [2.2–11.4]	4.6 [2.1–10.2]	7.3 [2.7–15.4]	0.016
D-dimer [mg/L]	1.5 [0.8–3.5]	1.5 [0.8–3.5]	2.3 [0.9–3.4]	0.554

**Table 3:** Spearman rank correlations (ρ) between clinical variables and mortality, mechanical ventilation, and ICU length of stay. The Pan score showed the strongest associations with mechanical ventilation (ρ = 0.49), ICU stay (ρ = 0.43), and mortality (ρ = 0.20). Age was primarily correlated with mortality (ρ = 0.23). CRP and erythrocyte count demonstrated weaker associations, whereas leukocyte count and D-dimer showed no meaningful correlation with any of the endpoints. Biological sex was coded as male = 1, female = 0; for this dichotomous variable, ρ corresponds to a rank-biserial association measure. Values in parentheses are *q*-values, i.e., *p*-values adjusted for multiple testing using the Benjamini–Hochberg false discovery rate procedure across all 21 tests reported in this table. Associations were considered statistically significant at *q* < 0.05. ICU, intensive care unit; CRP, C-reactive protein.

Variable	Death		Ventilation		ICU Stay	
	Correlation	*p*-Value (*q*-Value)	Correlation	*p*-Value (*q*-Value)	Correlation	*p*-Value (*q*-Value)
Age	0.23	<0.01 (<0.001)	0.01	0.911 (0.956)	−0.09	0.098 (0.172)
Biological sex	0.11	0.047 (0.099)	−0.02	0.644 (0.712)	0.07	0.220 (0.302)
Pan score [total mean]	0.20	<0.01 (<0.001)	0.49	<0.01 (<0.001)	0.43	<0.001 (<0.001)
Laboratory parameters						
Erythrocytes	−0.17	0.001 (0.006)	−0.10	0.070 (0.133)	−0.16	0.003 (0.011)
Leukocytes	0.03	0.589 (0.687)	0.06	0.230 (0.302)	0.07	0.177 (0.266)
CRP	0.14	0.019 (0.049)	0.12	0.034 (0.079)	0.17	0.004 (0.011)
D-dimer	0.05	0.554 (0.685)	0.12	0.140 (0.226)	−0.00	0.997 (0.997)

**Table 4:** Associations between the Pan score and clinical outcomes (logistic regression). Each one-point increase in the Pan score was associated with higher odds of mechanical ventilation as well as prolonged ICU stay. The estimate remained stable across all levels of adjustment. n denotes the analytic sample after listwise deletion and events the number of outcome events entering each model; the reduction in sample size in models including CRP and D-dimer reflects the incomplete availability of these parameters. The analytic sample for the ICU stay endpoint is 346 rather than 351 because the length of ICU stay was not documented for five patients. The model adjusted for age, sex and haematological parameters is the primary multivariable model, whereas models including CRP and D-dimer are reported as sensitivity analyses. All associations were statistically significant (*p* < 0.001). ICU, intensive care unit; CRP, C-reactive protein; OR, odds ratio.

Outcome	Model	*n*	Events	OR (95% CI)	*p*-Value
Mechanical ventilation	Unadjusted	351	137	1.28 (1.21–1.36)	<0.001
	+age, sex	351	137	1.29 (1.22–1.37)	<0.001
	+haematology (primary)	349	135	1.29 (1.22–1.38)	<0.001
	+CRP (sensitivity)	297	127	1.27 (1.19–1.36)	<0.001
	+D-dimer (sensitivity)	138	53	1.24 (1.13–1.37)	<0.001
ICU stay ≥ 7 days	Unadjusted	346	56	1.27 (1.19–1.36)	<0.001
	+age, sex	346	56	1.27 (1.19–1.36)	<0.001
	+haematology (primary)	344	55	1.27 (1.19–1.36)	<0.001
	+CRP (sensitivity)	292	48	1.28 (1.19–1.38)	<0.001
	+D-dimer (sensitivity)	137	15	1.26 (1.12–1.46)	<0.001

## Data Availability

The data sets (chest CT images) presented in this article are not readily available due to privacy and ethical restrictions.

## Supplementary Materials

The following supporting information can be downloaded at https://www.mdpi.com/article/10.3390/diagnostics16183008/s1. Table S1: Sensitivity analyses addressing heterogeneity in CT acquisition protocols; Table S2: Discriminatory performance of the Pan score for each reader and for the AI-based assessment; Table S3: Spearman rank correlations between clinical parameters and the mean Pan score, with adjusted *q*-values and the number of available observations.

## Abbreviations

The following abbreviations are used in this manuscript:

AI	Artificial intelligence
AUC	Area under the curve
CI	Confidence interval
COVID-19	Coronavirus disease 2019
CRP	C-reactive protein
CT	Computed tomography
df	Degrees of freedom
dL	Decilitre
eGFR	Estimated glomerular filtration rate
GGO	Ground-glass opacity
Hb	Haemoglobin
ICU	Intensive care unit
IQR	Interquartile range
i. v.	Intravenous
K	Potassium
L	Litre
LDH	Lactate dehydrogenase
LLL	Left lower lobe
LR	Likelihood ratio
LUL	Left upper lobe
mg	Milligramme
ML	Middle lobe
mL	Millilitre
mm	Millimetre
Na	Sodium
NIV	Non-invasive ventilation
nL	Nanolitre
OR	Odds ratio
PACS	Picture archiving and communication system
PCR	Polymerase chain reaction
PCT	Procalcitonin
pH	Potential of hydrogen
pL	Picolitre
pO_2_	Partial pressure of oxygen
PTT	Partial thromboplastin time
RIS	Radiological information system
RLL	Right lower lobe
ROC	Receiver operating characteristic
RUL	Right upper lobe
SARS-CoV-2	Severe acute respiratory syndrome coronavirus 2
SD	Standard deviation
SE	Standard error
WHO	World Health Organization
