# Supplementary material for: Evaluation of an Established Semi-Quantitative Chest CT Scoring System for Assessing the Severity of COVID-19 Pneumonia: What Is Its Diagnostic Value Regarding Patient Outcomes?
Source: Diagnostics (Basel). 2026 Sep 17;16(18):3008. doi: 10.3390/diagnostics16183008 (PMC13605799; doi:10.3390/diagnostics16183008)
Supplement: Supplementary file 1 [file diagnostics-16-03008-s001.zip › diagnostics-4423181-supplementary.pdf]

Table S1. Sensitivity analyses addressing heterogeneity in CT acquisition protocols.

Outcome	Model	n	Events	OR (95% CI)	p-value
**Mechanical ventilation**	Primary model	349	135	1.29 (1.22–1.38)	<0.001
	+ contrast medium	349	135	1.29 (1.22–1.38)	<0.001
	+ contrast medium + slice thickness	349	135	1.29 (1.22–1.38)	<0.001
	Non-contrast examinations only, unadjusted	55	27	1.34 (1.17–1.59)	<0.001
**ICU stay ≥ 7 days**	Primary model	344	55	1.27 (1.19–1.36)	<0.001
	+ contrast medium	344	55	1.26 (1.18–1.35)	<0.001
	+ contrast medium + slice thickness	344	55	1.27 (1.19–1.36)	<0.001
	Non-contrast examinations only, unadjusted	53	16	1.29 (1.14–1.52)	<0.001

The primary multivariable model was adjusted for age, sex, leukocyte count and erythrocyte count. Slice thickness was grouped as ≤1 mm, 2–3 mm (reference category), and 5 mm; the individual categories of 0.8 mm (n = 5) and 2 mm (n = 10) were too small to be analysed separately. The non-contrast subgroup comprises examinations performed for suspected COVID-19 pneumonia (n = 55); given the limited sample size, this analysis is reported unadjusted as a robustness check. Odds ratios refer to a one-point increase in the mean Pan score. n denotes the analytic sample after listwise deletion and events the number of outcome events. CI, confidence interval; ICU, intensive care unit; OR, odds ratio.

Table S2. Discriminatory performance of the Pan score for each reader and for the AI-based assessment.

Reader	Mortality	Mechanical ventilation	ICU stay ≥ 7 days
**Mean of all readers**	0.639 (0.565–0.713)	0.788 (0.739–0.837)	0.803 (0.733–0.873)
Radiology specialist	0.646 (0.574–0.718)	0.775 (0.724–0.826)	0.794 (0.722–0.865)
Assistant physician	0.569 (0.495–0.643)	0.751 (0.698–0.803)	0.773 (0.701–0.846)
Medical student	0.643 (0.569–0.717)	0.782 (0.732–0.831)	0.790 (0.721–0.860)
AI-based software	0.651 (0.578–0.725)	0.761 (0.710–0.812)	0.781 (0.712–0.849)
n / events	*351 / 75*	*351 / 137*	*346 / 56*

Areas under the receiver operating characteristic curve (AUC) with 95% confidence intervals are shown for all-cause mortality, mechanical ventilation, and prolonged ICU stay (≥ 7 days). "Mean of all readers" refers to the Pan score averaged across the three human readers and the AI-based assessment; this averaged score was used as the exposure variable in all main analyses. The final row gives the number of patients with complete data and the number of outcome events for each endpoint. AUC, area under the curve; CI, confidence interval; ICU, intensive care unit.

Table S3. Spearman rank correlations between clinical parameters and the mean Pan score, with adjusted q-values and the number of available observations.

Parameter	Spearman ρ	p-value	q-value	n
Ventilation hours	0.52	<0.001	<0.001	280
LDH	0.51	<0.001	<0.001	258
ICU length of stay	0.43	<0.001	<0.001	346
Interval from PCR to death	−0.37	0.001	0.004	71
CRP	0.29	<0.001	<0.001	297
PCT	0.28	<0.001	<0.001	180
PTT	0.25	<0.001	<0.001	276
O2 saturation	−0.24	<0.001	<0.001	268
pO2	−0.19	0.002	0.004	268
Prothrombin time (Quick)	−0.13	0.047	0.083	248
Erythrocytes	−0.12	0.024	0.050	349
Haemoglobin	−0.11	0.042	0.080	349
Age	−0.10	0.074	0.120	351
Sodium	−0.05	0.327	0.480	346
GFR	0.05	0.343	0.480	344
Potassium	0.04	0.484	0.636	340
Creatinine	−0.03	0.570	0.705	344
pH	0.02	0.712	0.786	268
Leukocytes	−0.02	0.702	0.786	349
D-dimer	0.01	0.930	0.977	155
Platelets	−0.00	0.992	0.992	349

Correlations are ordered by the absolute strength of the association. q-values are p-values adjusted for multiple testing using the Benjamini-Hochberg false discovery rate procedure across all 21 tests shown; associations were considered statistically significant at $q < 0.05$. n denotes the number of patients with complete data for the respective parameter. The interval from PCR confirmation to death is defined only for patients who died, which accounts for the small number of observations. These data underlie Figure 9. CRP, C-reactive protein; GFR, glomerular filtration rate; ICU, intensive care unit; LDH, lactate dehydrogenase; PCR, polymerase chain reaction; PCT, procalcitonin; PTT, partial thromboplastin time.
